# Alkaloid Extract of *Moringa oleifera* Lam. Exerts Antitumor Activity in Human Non-Small-Cell Lung Cancer via Modulation of the JAK2/STAT3 Signaling Pathway

**DOI:** 10.1155/2021/5591687

**Published:** 2021-06-08

**Authors:** Jing Xie, Lin-jie Peng, Ming-rong Yang, Wei-wei Jiang, Jia-ying Mao, Chong-ying Shi, Yang Tian, Jun Sheng

**Affiliations:** ^1^College of Food Science and Technology, Yunnan Agricultural University, Kunming 650224, China; ^2^Engineering Research Center of Development and Utilization of Food and Drug Homologous Resources, Ministry of Education, Yunnan Agricultural University, Kunming 650224, China; ^3^National Research and Development Professional Center for Moringa Processing Technology, Yunnan Agricultural University, Kunming 650224, China; ^4^Key Laboratory of Pu-er Tea Science, Ministry of Education, Yunnan Agricultural University, Kunming 650224, China

## Abstract

Lung cancer is one of the most common malignant tumors diagnosed worldwide. *Moringa oleifera* Lam. is a valuable medicinal plant native to India and Pakistan. However, the antilung cancer activity of *M. oleifera* alkaloid extract (MOAE) is unknown. The present study aimed to evaluate the regulatory effect of MOAE on A549 cells by examination of the proliferation, apoptosis, cell cycle, and migration of cells and to elucidate the possible mechanism of action of MOAE. We tested five types of cancer cells and four types of lung cancer cells and found MOAE exerted the strongest growth inhibitory effect against A549 cells but had low toxicity to GES-1 cells (human gastric mucosal epithelial cells). Simultaneously, MOAE induced apoptosis and increased the expression of the apoptosis-related proteins caspase-3 and caspase-9 in A549 cells. Furthermore, MOAE induced cell cycle arrest in the S phase through a decrease in the expression of the proteins cyclin D1 and cyclin E and an increase in the expression of the protein p21. MOAE also inhibited the migratory ability of A549 cells and decreased the expression of the migration-related proteins, matrix metalloproteinase (MMP) 2 and MMP9. In addition, the phosphorylation level of JAK2 and STAT3 proteins was decreased in MOAE-treated A549 cells. Furthermore, AZD1480 (a JAK inhibitor) and MOAE inhibited the proliferation and migration of A549 cells and induced cell apoptosis, and the effects of MOAE and AZD1480 were not additive. These results indicated that MOAE inhibits the proliferation and migration of A549 cells and induces apoptosis and cell cycle arrest through a mechanism that is related to the inhibition of JAK2/STAT3 pathway activation. Thus, this extract has potential for preventing and treating lung cancer.

## 1. Introduction

Non-small-cell lung cancer (NSCLC) comprises approximately 80% of cancers globally and is associated with the highest morbidity and mortality of all malignant tumors [1, 2]. Despite the progress achieved through the optimization of surgical methods, adjuvant therapy, and comprehensive multimodal therapy, the treatment outcomes and prognosis of patients with NSCLC, especially those with advanced disease, remain unsatisfactory. In addition, existing treatments and drugs are accompanied by serious adverse reactions that significantly reduce the quality of life of the patients [3]. Therefore, to improve the effects of therapy and to increase overall patient survival, new agents with minimal side effects and good treatment efficacy must be identified.

*Moringa oleifera* Lam. (Moringaceae), also known as the drumstick tree, is a tropical and subtropical plant known colloquially as the “miracle tree” owing to its rich nutritional and medicinal value. *M. oleifera* extracts possess anti-inflammatory [4], cardioprotective [5], hypocholesterolemic [6], antidiabetic [7], antihypertensive [8, 9], neuroprotective [10], hepatoprotective [11], antioxidant [12], and antibacterial properties [13], among others. Additionally, various parts of the *M. oleifera* plant, including the leaves, stem bark, fruit pods, and seeds, have growth inhibitory effects in various types of tumor cells, including lung cancer [14], liver cancer [15], leukemia [16], pancreatic cancer [17], and cervical cancer [18].

Alkaloids are a class of nitrogen-containing organic alkaline compounds with analgesic, spasm-relieving, antibacterial, anti-inflammatory, hypotensive, antiasthmatic, and antitumoral properties [19]. As many alkaloids are high-efficiency and low-toxicity antitumoral compounds, their potential use as cancer treatment has attracted the attention of researchers worldwide. More than 200 compounds have been isolated from the flowers, roots, leaves, and fruits of *M. oleifera*. Among them, the alkaloids *N*, *α*-L-rhamnopyranosyl vincosamide, *N*-benzylcarbamic acid, deoxy-niazimicin, 3-dibenzyl urea, and aurantiamide acetate have been reported to exert protective effects against cardiovascular diseases and to participate in the removal of free radicals [5, 20, 21]. However, the antilung cancer activity of *M. oleifera* alkaloids has not been confirmed.

The aim of the present study was to evaluate the regulatory effect of *M. oleifera* alkaloid extract (MOAE) on NSCLC A549 cells by evaluating the proliferation, apoptosis, cell cycle, and migration of cells and to elucidate the possible mechanism of action of MOAE.

## 2. Materials and Methods

### 2.1. Separation and Extraction of *M. oleifera* Alkaloids

Alkaloids were prepared as described previously [22]. In brief, *M. oleifera* leaf powder (10 kg; Yunnan Tianyou Technology Development Co., Ltd., Dehong, Yunnan, China) was extracted three times with 50% ethanol, and the extracts were filtered and combined. The combined ethanol extracts were then concentrated using a rotary evaporator at 50°C. The concentrated extracts were adjusted to pH 2 by the addition of 10% HCl and extracted three times with ethyl acetate. The extracts were combined and concentrated with a rotary evaporator to remove ethyl acetate. The acidic aqueous solution was adjusted to pH 10 with sodium hydroxide and then extracted three times with chloroform. The extracts were combined and concentrated with a rotary evaporator to remove chloroform; finally, 30 g of alkaloid extract was obtained.

### 2.2. LTQ-Orbitrap High-Resolution Mass Spectrometry Analysis of MOAE

The composition of the MOAE was analyzed using an LTQ-Orbitrap linear ion trap-tandem electrostatic field orbital trap high-resolution mass spectrometer (Thermo Fisher, Waltham, MA, USA) equipped with an FTZ Orbitrap mass detector and an electrospray ion source (ESI). The mass scanning range was 100–1,000 *m/z*, the spray voltage was 4.0 kV, the tubular lens voltage was 110 V, the capillary temperature was 350°C, the sheath gas flow was 30 L/h, and the auxiliary gas flow was 10 L/h. The instrument was operated in positive mode, and the resolution was set to 30,000. Data-dependent scanning was used for the analysis of secondary and tertiary peaks, and the three peaks with the highest abundance were selected for collision-induced dissociation (CID) and fragment scanning. Fragment ions were detected using an ion trap.

### 2.3. Cell Culture

A549, HCT116, A375, MDA-MB-231, Hep-G2, NCI-H1975, NCI-H1781, NCI-H441, and GES-1 cells were purchased from the Kunming Institute of Zoology, Chinese Academy of Sciences. The cells were cultured in DMEM/F12 medium, RPMI 1640 medium, or DMEM high-glucose medium (HyClone, CA, USA), as appropriate, containing 10% fetal bovine serum (HyClone), 1% of 1 g/mL streptomycin, and 1000 U/mL penicillin (Solarbio, Beijing, China), at 37°C in a humidified incubator with an atmosphere containing 5% CO_2_.

### 2.4. MTT Assay

A549, HCT116, A375, MDA-MB-231, and Hep-G2 cells in the logarithmic growth phase were seeded in 96-well plates (1 × 10^4^ cells/well), cultured at 37°C in a humidified incubator with an atmosphere containing 5% CO_2_ for 12–24 h, and then treated with different concentrations of MOAE (0, 25, 50, 100, 200, or 400 µg/mL) for 48 h. The control cells were treated with 0.1% DMSO. After 48 h, the supernatant was removed, and 100 *µ*L of MTT solution (0.25 mg/mL) was added to each well. After incubation for 4 h, the supernatant was removed and 200 *µ*L of DMSO was added to each well. The cells were shaken for 10 min to ensure complete dissolution of the purple crystals, and the absorbance at 490 nm was then measured. The IC_50_ value (the concentration of the drug capable of causing 50% inhibition of cell survival) was calculated using SPSS software.

A549, NCI-H1975, NCI-H1781, NCI-H441, and GES-1 cells (1 × 10^4^ cells/well) were seeded in 96-well plates for 24 h and treated with MOAE (0, 100, 200, 400, or 800 *µ*g/mL) for 24 h or 48 h. After 24 h and 48 h, the cell viability was evaluated by the MTT assay.

A549 cells (1 × 10^4^ cells/well) were seeded in 96-well plates for 24 h. The cells were pretreated with the JAK inhibitor AZD1480 (2.5 *µ*M) (Selleck Chemicals, Houston, TX, USA) for 2 h, then treated with MOAE (0 or 200 µg/mL) for 48 h. After 48 h, the cell viability was evaluated by the MTT assay.

### 2.5. Observation of Cell Morphology

A549 cells were seeded in 60 mm culture plates at a density of 1 × 10^6^ cells/plate and left to adhere. MOAE (0, 100, or 200 *µ*g/mL) treatment was applied for 48 h. The supernatant was then removed, fresh culture medium was added, and cell morphology was observed under an inverted microscope.

### 2.6. Colony Formation Assay

The colony formation assay was conducted as previously described [23]. A549 cells were seeded in 6-well plates at a density of 500 cells/well, cultured at 37°C in a humidified incubator with an atmosphere containing 5% CO_2_ for 12–24 h, and then treated with different concentrations of MOAE (0, 100, or 200 *µ*g/mL) for 48 h. Subsequently, the culture medium was replaced with fresh medium, and the cells were cultured for a further 15 days. Then, the cells were fixed in methanol and stained with 0.1% crystal violet for 15 min. After staining, the cells were washed several times with PBS, and the plates were air-dried and imaged. The crystal violet stain was dissolved in 10% glacial acetic acid, and the absorbance at 560 nm was measured.

### 2.7. Cell Apoptosis and Cell Cycle Analysis

Flow cytometry was performed as previously described [24]. To analyze apoptosis, A549 cells were first seeded in 6-well plates (1 × 10^6^ cells/plate) and treated with MOAE (0, 100, or 200 *µ*g/mL) for 48 h. Then, the cells were collected, washed twice with precooled PBS, and centrifuged. Binding buffer (100 *µ*L) containing 5 *µ*L of Annexin V/fluorescein isothiocyanate (FITC) and 10 *µ*L of 20 mg/mL propidium iodide (PI; Sigma–Aldrich, Germany) was added to the cells, which were incubated for 10–15 min and then analyzed by flow cytometry (BD FACSCalibur, CA, USA) within 1 h.

A549 cells (1 × 10^6^ cells/plate) were seeded in 6-well plates for 24 h. The cells were pretreated with the JAK inhibitor AZD1480 (2.5 *µ*M) for 2 h and then treated with MOAE (0 or 200 *µ*g/mL) for 48 h. After 48 h, cell apoptosis was measured as described above.

For cell cycle analysis, A549 cells were harvested as above, washed twice with precooled PBS, fixed in 70% ethanol, and placed in a refrigerator at 4°C overnight. After washing with PBS, 500 *μ*L of RNase/PI dye was added to the cells, which were incubated for 30 min and then analyzed by flow cytometry within 1 h.

### 2.8. Western Blotting Assay

Western blotting was performed as previously described [25]. A549 cells were seeded in 60 mm culture plates (1 × 10^6^ cells/plate) and treated with MOAE (0, 100, or 200 *µ*g/mL) for 48 h. Total protein was extracted from A549 cells using RIPA buffer containing phenylmethylsulfonyl fluoride (PMSF) (RIPA : PMSF = 100 : 1). The proteins were separated by 10% SDS–PAGE, transferred to polyvinylidene fluoride (PVDF) membranes, and then blocked with 5% skimmed milk powder at room temperature for 1 h to prevent nonspecific binding. The membranes were incubated overnight at 4°C with primary antibodies against caspase-3, caspase-9, cyclin D1, cyclin E (Santa Cruz mouse monoclonal antibodies; catalog numbers sc-7272, sc-56076, sc-8396, and sc-247, respectively; all 1 : 1000 dilution), p21, MMP2, MMP9 (Abcam rabbit monoclonal antibodies; catalog numbers ab109520, ab92536, and ab76003, respectively; all 1 : 2000 dilution), JAK2, p-JAK2, STAT3, p-STAT3, and *β*-actin (Cell Signaling Technology rabbit monoclonal or polyclonal antibodies; catalog numbers 12640, 9145, 3230, 3771, and 4970, respectively; 1 : 2000 dilution). The membranes were washed three times with PBST and incubated with horseradish peroxidase-conjugated goat anti-rabbit/anti-mouse secondary antibody (1 : 5,000; R&D Systems, USA) for 1 h. After a further three washes with PBST, the protein bands were detected by chemiluminescence and analyzed using ImageJ and GraphPad Prism 5.

### 2.9. Wound Healing Assay

A549 cells (1 × 10^6^ cells/plate) were seeded in 60 mm culture plates and allowed to adhere. A sterile pipette tip was used to generate a scratch across the cell layer, which was then imaged using an inverted microscope [26]. The cells were subjected to treatment (either pretreatment with the JAK inhibitor AZD1480 (2.5 *µ*M) for 2 h and then treatment with MOAE (0 or 200 *µ*g/mL) or only MOAE (0 or 200 *µ*g/mL)) for 48 h and imaged again. The results were analyzed using ImageJ and GraphPad Prism 5.

### 2.10. Transwell Migration Assay

Cell migration through Transwell filters was analyzed as previously described [27]. The migratory ability of A549 cells was examined using 24-well Transwell plates with 8 *µ*m pore membrane inserts (Corning, NY, USA). Cell suspensions (200 *µ*L (2 × 10^4^ cells) in serum-free medium) containing different concentrations of MOAE (0, 100, or 200 *µ*g/mL) were added to the upper chamber of the Transwell plates. Then, 600 *µ*L of complete medium containing 10% FBS was added to the lower chamber, and the cells were incubated at 37°C for 48 h. Noninvading cells in the upper chamber were removed with a cotton swab. The migrated cells were fixed in methanol, stained with crystal violet, and observed and photographed under a microscope. The number of migrated cells was counted using a hemocytometer.

### 2.11. Statistical Analysis

Data presented as bar graphs show the mean ± standard error of the mean (SEM) of at least three independent experiments. The statistical significance of data was evaluated using one-way ANOVA analysis of variance by GraphPad software. For data not conforming to a normal distribution and equal variance, nonparametric tests were used. *p* values of <0.05, <0.01, or <0.001 were considered to indicate a statistically significant difference.

## 3. Results

### 3.1. MOAE Inhibited the Proliferation of A549 Cells

**Figure 1:**
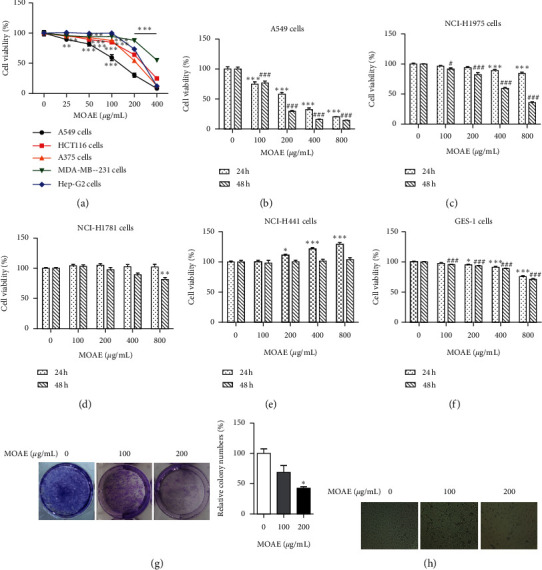
MOAE inhibits the proliferation of A549 cells. (a) The relative viability of A549, A375, HCT116, Hep-G2, and MDA-MB-231 cells following MOAE (0–400 *μ*g/mL) treatment for 48 h. ^*∗∗*^*p* < 0.01 and ^*∗∗∗*^*p* < 0.001*vs*. 0 *μ*g/mL. The relative cell viability of A549 (b), NCI-H1975 (c), NCI-H1781 (d), NCI-H441 (e), and GES-1 (f) cells following MOAE (0–800 *μ*g/mL) treatment for 24 h or 48 h. ^*∗*^*p* < 0.05, ^*∗∗*^*p* < 0.01, and ^*∗∗∗*^*p* < 0.001*vs*. 0 *μ*g/mL at 24 h; *^#^p* < 0.05 and *^###^p* < 0.001 vs. 0 *μ*g/mL at 48 h. (g) The relative number of colonies of A549 cells after MOAE treatment (0, 100, or 200 *μ*g/mL) for 48 h. ^*∗*^*p* < 0.05*vs*. 0 *μ*g/mL. (h) The morphological changes in A549 cells after treatment with different concentrations of MOAE (0, 100, or 200 *μ*g/mL) for 48 h. The results are expressed as the mean ± SEM of three independent experiments.

To investigate the antitumor activity of MOAE, we evaluated the growth inhibitory effect of MOAE on A549, A375, HCT116, MDA-MB-231, and Hep-G2 cells by the MTT assay. MOAE significantly inhibited the growth of A549 cells in a concentration-dependent manner. The IC_50_ of MOAE in A549, A375, HCT116, Hep-G2, and MDA-MB-231 cells was 158.67 *μ*g/mL, 238.61 *μ*g/mL, 276.96 *μ*g/mL, 283.07 *μ*g/mL, and 413.13 *μ*g/mL, respectively (Figure 1(a)). As a further investigation of the effect of MOAE on the growth of different lung cancer cells, A549, NCI-H1975, NCI-H1781, and NCI-H441 cells were treated with MOAE (0, 100, 200, 400, or 800 *μ*g/mL) for 24 h or 48 h. Compared with the control, MOAE significantly inhibited the proliferation of A549 and NCI-H1975 cells in a concentration- and time-dependent manner (Figures 1(b) and 1(c)). MOAE had the most significant inhibitory effect on A549 cells. In addition, MOAE was found to have no inhibitory effect on the growth of NCI-H1781 and NCI-H441 cells (Figures 1(d) and 1(e)). To determine whether MOAE was toxic to normal cells, we evaluated the effect of MOAE on the growth of GES-1 cells by the MTT assay. We found that for an MOAE dose of 800 *μ*g/mL, the cell survival percentage at 24 h and 48 h was 75.76% and 71.15%, respectively (Figure 1(f)). These results indicated that MOAE had the strongest growth inhibitory effect on A549 cells but had low toxicity to GES-1 cells.

In addition, at 48 h, MOAE treatment also reduced the clone-formation rate of A549 cells compared with the control group, from 100% ± 7.37% (0 *µ*g/mL) to 68.69% ± 11.23% and 42.57% ± 2.39% (100 and 200 *µ*g/mL MOAE, respectively) (Figure 1(g)). Moreover, unlike the control cells that were polygonal in shape and tightly arranged, MOAE-treated A549 cells were loosely arranged and were further apart, and some cells were observed to shrink and dissolve (Figure 1(h)). Collectively, these results showed that MOAE could inhibit the growth of A549 cells.

### 3.2. MOAE Induced A549 Cell Apoptosis

**Figure 2:**
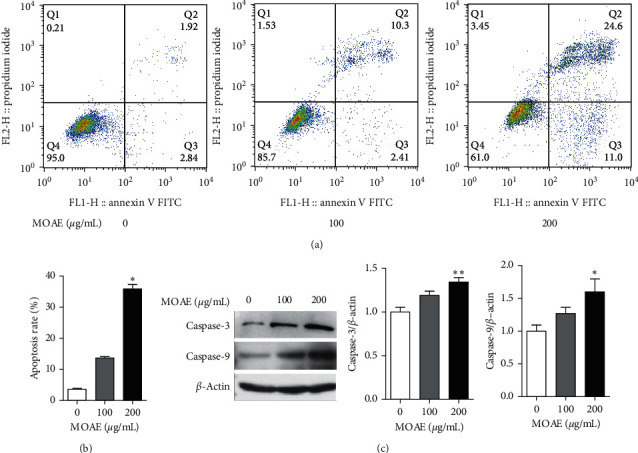
MOAE induces apoptosis in A549 cells. A549 cells were treated with MOAE (0, 100, or 200 *μ*g/mL) for 48 h. (a) The percentage of apoptotic cells was determined by flow cytometry. (b) The percentage of apoptotic cells in each treatment group. (c) The expression of the apoptosis-related proteins caspase-3 and caspase-9 in A549 cells was measured by western blotting assay, with *β*-actin used as the loading control. Quantification of the relative levels of caspase-3 and caspase-9 proteins. The results are expressed as the mean ± SEM of three independent experiments. ^*∗*^*p* < 0.05 and ^*∗∗*^*p* < 0.01*vs*. 0 *μ*g/mL.

As MOAE-treated A549 cells exhibited apoptotic characteristics, we assessed cell apoptosis by flow cytometry. As shown in Figures 2(a) and 2(b), compared with the control cells (3.62% ± 0.24%), the percentage of apoptotic cells was higher following 100 and 200 *µ*g/mL MOAE treatment at 13.63% ± 0.43% and 35.89% ± 1.38%, respectively. Furthermore, MOAE treatment at 200 *µ*g/mL led to a significant increase in the expression of the apoptosis marker proteins caspase-3 (*p* < 0.01) and caspase-9 (*p* < 0.05) (Figure 2(c)). These results indicated that MOAE induced caspase-dependent apoptosis in A549 cells.

### 3.3. MOAE Induced Cell Cycle Arrest

**Figure 3:**
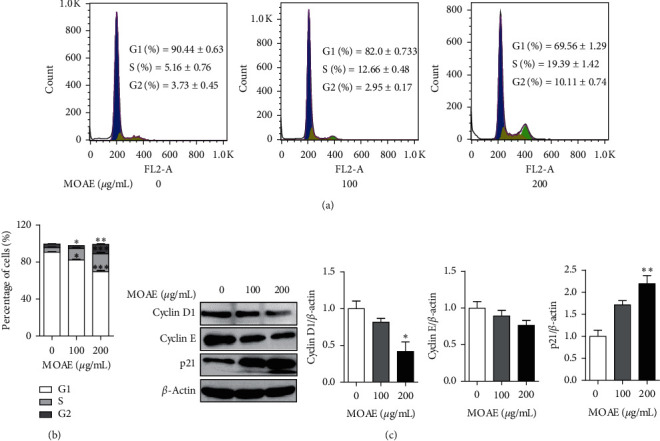
MOAE induces cell cycle arrest in A549 cells. A549 cells were treated with MOAE (0, 100, or 200 *μ*g/mL) for 48 h. (a) The cell cycle of A549 cells was analyzed by flow cytometry; (b) statistical analysis of three independent experiments. (c) The expression of the cell cycle-related proteins cyclin D1, cyclin E, and p21 in A549 cells was assessed by western blotting assay, with *β*-actin used as the loading control. Quantification of the relative levels of the proteins cyclin D1, cyclin E, and p21. The results are expressed as the mean ± SEM of three independent experiments. ^*∗*^*p* < 0.05, ^*∗∗*^*p* < 0.01, and ^*∗∗∗*^*p* < 0.001*vs*. 0 *μ*g/mL.

To determine the effect of MOAE on the cell cycle, we examined the cell cycle of A549 cells by flow cytometry. The proportion of cells in the G1 phase was significantly reduced, and the proportion of cells in the S phase was significantly increased in a MOAE concentration-dependent manner. The percentage of A549 cells in the G1 phase decreased from 90.44% ± 0.63% (0 *µ*g/mL) to 82.03% ± 0.73% and 69.56% ± 1.29%, respectively, whereas the percentage of A549 cells in the S phase increased from 5.16% ± 0.76% to 12.66% ± 0.48% and 19.39% ± 1.42%, respectively, after treatment with MOAE (100 and 200 *µ*g/mL) (Figures 3(a) and 3(b)). We then measured the expression levels of cell cycle-related proteins by western blotting. As shown in Figure 3(c), compared with the control group, MOAE treatment at 200 *µ*g/mL decreased the expression of cyclin D1 (*p* < 0.05) and cyclin E but increased the expression of p21 (*p* < 0.01) (Figure 3(c)). These results indicated that MOAE induced cell cycle arrest in A549 cells and modulated the expression of cell cycle-related proteins.

### 3.4. MOAE Inhibited A549 Cell Migration

**Figure 4:**
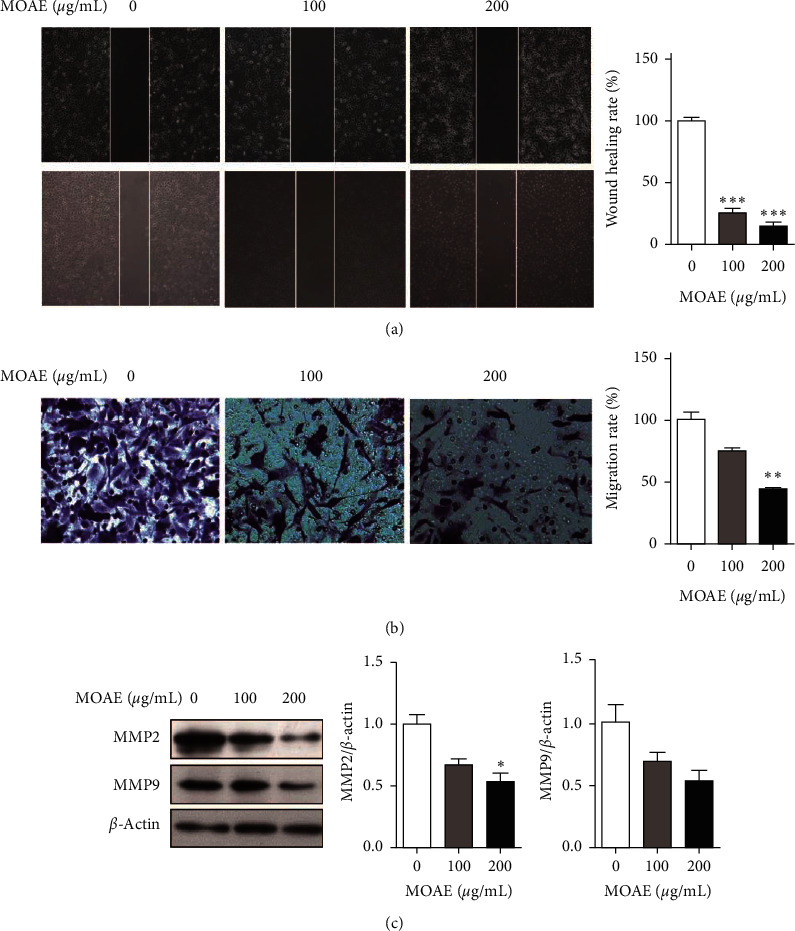
MOAE inhibits migration of A549 cells. A549 cells were treated with MOAE (0, 100, or 200 *μ*g/mL) for 48 h. The cell migration was assessed by (a) wound-healing and (b) Transwell migration assays. (c) The expression of the migration-related proteins MMP2 and MMP9 was measured by western blotting assay, with *β*-actin as the loading control. The results are expressed as the mean ± SEM of three independent experiments. ^*∗*^*p* < 0.05, ^*∗∗*^*p* < 0.01, and ^*∗∗∗*^*p* < 0.001 vs. 0 *μ*g/mL.

To investigate the inhibitory effect of MOAE on the migratory ability of A549 cells, we analyzed the migration rate of cells by wound healing and Transwell migration assays. Wound healing in A549 cells was significantly inhibited by MOAE in a concentration-dependent manner. Compared with the control group (100% ± 3.01%), the wound-healing rate decreased to 25.45% ± 3.75% and 15.18% ± 2.74%, following MOAE treatment at 100 *µ*g/mL and 200 *µ*g/mL, respectively (Figure 4(a)). Moreover, the cell migration rate was decreased from 100% ± 5.89% to 74.60% ± 2.60% and 44.41% ± 0.42%, respectively (Figure 4(b)). Western blotting assay results showed that, compared with controls, MOAE treatment at 200 *µ*g/mL inhibited the expression of the cell migration-related proteins MMP2 (*p* < 0.05) and MMP9 in A549 cells in a concentration-dependent manner (Figure 4(c)). Together, these results indicated that MOAE could inhibit A549 cell migration by regulating the expression of migration-related proteins.

### 3.5. MOAE Inhibited the Activation of the JAK2/STAT3 Signaling Pathway in A549 Cells

**Figure 5:**
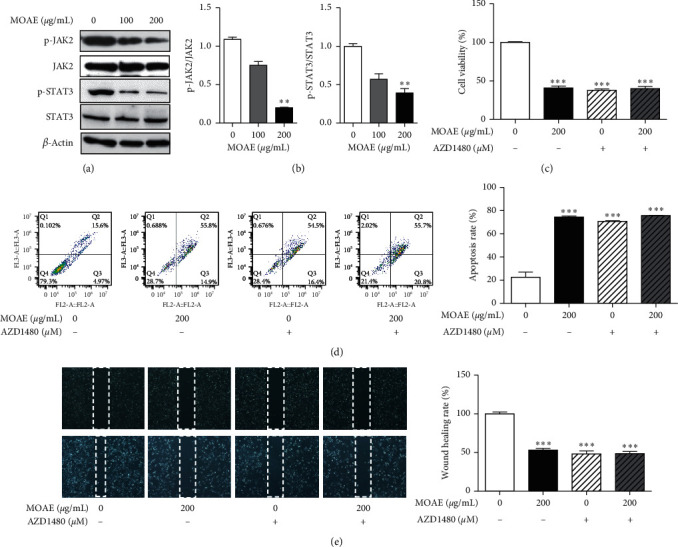
MOAE inhibits the activation of the JAK2/STAT3 signaling pathway in A549 cells. A549 cells were treated with MOAE (0, 100, or 200 *μ*g/mL) for 48 h. (a) The protein expression of JAK2, p-JAK2, STAT3, and p-STAT3 was measured by western blotting assay, with *β*-actin used as the loading control. (b) Quantification of the relative levels of p-JAK2 and p-STAT3; each value was normalized to that of JAK2 and STAT3, respectively. Cell viability (c), cell apoptosis (d), and cell migration (e) were analyzed using the MTT assay, flow cytometry, and wound healing assay, respectively. A549 cells were pretreated for 2 h with AZD1480 (2.5 *μ*M) before treatment with MOAE (0 or 200 *μ*g/mL) for 48 h. The results are expressed as the mean ± SEM of three independent experiments. ^*∗∗*^*p* < 0.01 and ^*∗∗∗*^*p* < 0.001*vs*. 0 *μ*g/mL.

Studies have shown that the Janus kinase 2/signal transducer and activator of transcription 3 (JAK2/STAT3) signaling pathway is overactive in NSCLC tissues and is closely associated with proliferation, angiogenesis, invasion, and migration of NSCLC cells [28]. To verify the inhibitory effect of MOAE on the JAK2/STAT3 signaling pathway in A549 cells, we evaluated the protein expression of JAK2, p-JAK2, STAT3, and p-STAT3 by western blotting assay. As shown in Figures 5(a) and 5(b), MOAE (200 *µ*g/mL) significantly inhibited the expression of p-JAK2 (*p* < 0.01) and p-STAT3 (*p* < 0.01) in a concentration-dependent manner. To determine the specific roles of JAK in the MOAE-mediated inhibition of cell growth and migration and induction of apoptosis, the cells were pretreated for 2 h with AZD1480 (a JAK inhibitor). Consistent with previous results, a significant decrease in cell viability and migration and an increase in apoptosis were observed in MOAE-treated A549 cells. Similarly, after pretreatment with AZD1480, cell viability and migration were significantly decreased, and apoptosis was significantly increased. However, AZD1480 and MOAE had no synergistic effect on proliferation, migration, and apoptosis in A549 cells (Figures 5(c)–5(e)). These results indicated that MOAE inhibits cell proliferation and migration and induces cell apoptosis through inhibition of the activation of JAK2/STAT3 signaling pathway.

### 3.6. Analysis of the Chemical Constituents of MOAE Using LTQ-Orbitrap Mass Spectrometry

**Figure 6:**
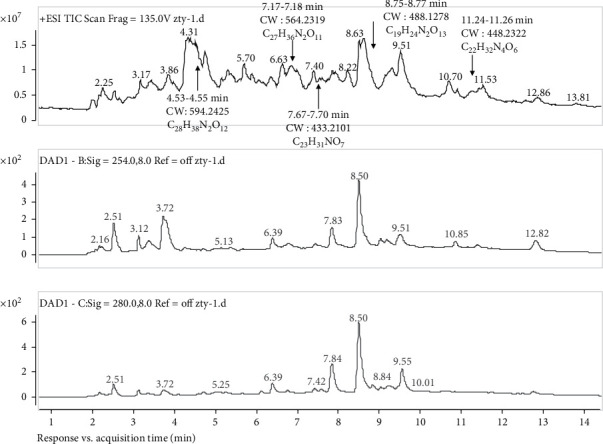
Analysis of the chemical constituents of MOAE using LTQ-Orbitrap mass spectrometry (MS). Total ion chromatogram of MOAE.

Five compounds (C_28_H_38_N_2_O_12_, C_27_H_36_N_2_O_11_, C_23_H_31_NO_7_, C_19_H_24_N_2_O_13_, and C_22_H_32_N_4_O_6_) were identified using LTQ-Orbitrap high-resolution mass spectrometry. The retention times of the five compounds were 4.53–4.55, 7.17–7.18, 7.67–7.70, 8.75–8.77, and 11.24–11.26 min, respectively. The molecular masses of these analytes were 594.2425, 564.2319, 433.2101, 488.1278, and 448.2322 g/mol, respectively (Figure 6).

## 4. Discussion

*M. oleifera* possesses a variety of pharmacological properties; the most prominent is its antitumor activity. For example, the water extract of *M. oleifera* leaves is known to inhibit growth and induce apoptosis in lung cancer [14], liver cancer [15], oral cancer [29], pancreatic cancer [17], esophageal cancer [30], Ehrlich ascites carcinoma [31], and human melanoma cells [32]. The alcohol extract of *M. oleifera* leaves can inhibit cell growth and induce apoptosis and cell cycle arrest in breast cancer, colon cancer [33], leukemia [16], and cervical cancer [18] cells. The methanolic extract of *M. oleifera* leaves can inhibit cell growth and induce apoptosis and cell cycle arrest in cervical cancer [34] and prostate cancer [35] cells. Similarly, the phenolic extract of *M. oleifera* leaves can induce apoptosis in human melanoma cells [36]. In addition, the bark and seeds of *M. oleifera* also have antitumor activity [33, 37, 38]. These results demonstrated that *M. oleifera* has good antitumor activity against a variety of cancers; however, studies of *M. oleifera* have mainly assessed the antitumor activity of the crude extract.

As numerous studies have confirmed that alkaloids may be the main mediators of the antitumor activity of many plants, we extracted and prepared MOAE for the study of its anticancer activity. In this study, we found that MOAE caused a certain degree of inhibition on the growth of A549 cells, A375 cells, HCT116 cells, MDA-MB-231 cells, and Hep-G2 cells, with the strongest inhibitory activity in A549 cells. Of the four types of lung cancer cells tested, MOAE selectively inhibited the growth of A549 cells. In addition, we found that MOAE treatment could inhibit migration in A549 cells as well as induce apoptosis and cell cycle arrest. We also found that the underlying mechanism may be related to the inhibition of JAK2/STAT3 signaling pathway activation.

Hyperproliferation and blocked apoptosis are among the main features of tumor cells that play an important role in cancer occurrence and development. Consequently, inhibiting tumor cell proliferation and inducing apoptosis are the main strategies for tumor treatment. Caspase-3 and caspase-9 are the major inducers of cell apoptosis through the promotion of DNA degradation and the formation of apoptotic bodies [39]. In this study, we found that MOAE treatment inhibited the proliferation of A549 cells in a concentration- and time-dependent manner. In addition, MOAE significantly induced apoptosis in A549 cells and increased the expression of caspase-3 and caspase-9.

An abundance of studies has found that *M. oleifera* can induce cell cycle arrest in various tumor cell types. For example, *M. oleifera* leaves and bark extracts induced significant G2/M phase arrest in MDA-MB-231 breast cancer cells and HCT-8 colon cancer cells [33]; water extracts of *M. oleifera* leaves induced cell cycle arrest in Hep-G2 cells by reducing the ratio of cells in the G1, S, and G2/M phases [40]; *M. oleifera* isothiocyanate [4-(*α*-L-rhamnopyranosyloxy)benzyl C] induced cell cycle arrest in human neuroblastoma SH-SY5Y cells by increasing the populations of cells in the G2 and S phase, decreasing the population of cells in the G1 phase, and increasing the expression of the protein p21 [41]; Hep-G2 cells treated with *M. oleifera* diethyl ether extracts and ethyl acetate extracts were arrested in the G2/M phase [42]; and finally, a leaf extract of *M. oleifera* induced cell cycle arrest in Panc-1 pancreatic cancer cells by increasing the population of cells in the sub-G1 phase [43]. In our study, MOAE treatment reduced the proportion of cells in the G1 phase and increased the number of cells in the S phase, while decreasing the expression of cyclin D1 and cyclin E and increasing the expression of p21. Together, these results showed that MOAE could induce cell cycle arrest in A549 cells.

Tissue infiltration and distant metastasis are two other important biological phenotypes of tumor cells [44]. The cause of death of most patients with NSCLC is not the primary lesions but the subsequent tumor metastases [45]. The MMPs are zinc ion-dependent endopeptidases that can degrade the extracellular matrix and vascular basement membrane; they play an important role in the invasion and metastasis of tumor cells by helping them to break through the basement membrane barrier [46]. Among the various members of the MMP family, MMP2 and MMP9 have major roles in cancer metastasis. Several studies have shown that inhibiting the expression of MMP2 or MMP9 can suppress the migration of A549 cells. For example, isolinderalactone inhibits A549 cell migration by downregulating MMP2 expression [47], whereas the ethanol extract of the leaves of *Dillenia pentagyna* suppresses the migration of A549 cells by decreasing the expression of MMP2 and MMP9 [48]. In addition, angelicin reduces the migratory capacity of A549 cells by reducing the expression of MMP2 and MMP9 [49]. In this study, we found that MOAE inhibited the migratory ability of A549 cells and decreased the protein expression of MMP2 and MMP9 in A549 cells.

The treatment strategies of NSCLC include surgery, radiation, chemotherapy, targeted therapy, or immunotherapy, either alone or in combination. However, approximately 80% of patients with NSCLC still develop stage IV tumors, and the 5-year relative survival rate is less than 20%. This may be related to tumor resistance during the treatment process, that is, the tumor's response to tyrosine kinase inhibitors (TKIs) or immune checkpoint blockers (ICBs) becomes impeded. Consequently, researchers have focused on alternative druggable targets in NSCLC to provide new therapies or improve existing treatments [50]. The JAK2/STAT3 signaling pathway is frequently activated in NSCLC and regulates a variety of cell functions, including proliferation, cell differentiation, metastasis, angiogenesis, apoptosis, and immune response; hence, STAT3 and its upstream activator JAK1/2 are considered promising targets [50–52]. Concurrently, studies have shown that STAT3 signaling mediates the resistance of NSCLC to EGFR-targeted therapies [53]. Therefore, the inhibition of JAK2/STAT3 signal transduction is an effective strategy for NSCLC treatment. At present, studies have demonstrated that numerous phytochemicals can interfere with the JAK/STAT signaling mechanism in human malignant cells, including phenolics, polyphenols, terpenoids, alkaloids, saponins, steroids, lignans, and phytoalexins [54]. Therefore, phytochemicals are a potential lung cancer treatment. In the present study, we found that MOAE decreased the levels of JAK2 and STAT3 phosphorylation in A549 cells, indicating that MOAE could suppress the activation of the JAK2/STAT3 signaling pathway. Furthermore, AZD1480 inhibited the proliferation and migration of A549 cells, and the effects of MOAE and AZD1480 were not additive, indicating that MOAE inhibited the proliferation and migration of A549 cells by suppressing the JAK2/STAT3 signaling pathway.

Although the current targeted therapies and personalized therapies for cancer have achieved remarkable success, the genetic heterogeneity of tumors, high costs, and high toxicity of treatments, among other issues, have severely restricted the treatment of cancer. Therefore, to solve these problems, scientists have proposed the concept of a low-toxicity “broad-spectrum” therapeutic approach [55]. This broad-spectrum treatment method involves the combination of a variety of low-toxicity drugs, including the use of plant- and food-derived chemicals that have been studied or used for cancer prevention and treatment. These combinations can collectively affect many pathways that are critical to the occurrence and spread of cancer. As these natural products are highly efficient and have low toxicity and low cost, they are widely favored. For example, it has been confirmed that many natural products, including resveratrol, epigallocatechin gallate (EGCG), curcumin, and lycopene, can induce tumor cell apoptosis and cell growth arrest. In addition, these natural products can be combined with synthetic drugs to exert synergistic effects; thus, they have potential clinical applications. *M. oleifera* leaves are eaten as a vegetable in countries such as China and India. In this study, we have confirmed that MOAE inhibits the growth and migration of A549 cells and does not strongly effect on the growth of GES-1 cells. Therefore, MOAE derived from edible plants is a candidate substance for the broad-spectrum therapeutic approach, which has the potential to be used in combination treatments.

Five alkaloids isolated from the leaves, stems, seeds, and roots of *M. oleifera*—*N*, *α*-L-rhamnopyranosyl vincosamide, *N*-benzylcarbamic acid, deoxy-niazimicin, 1, 3-dibenzyl urea, and aurantiamide acetate—exert protective effects against cardiovascular disease and can remove free radicals [5, 20, 21]. In this study, despite high-resolution mass spectrometric analysis of MOAE, we were unable to identify some of the highly abundant compounds present in the extract; therefore, further systematic analysis is needed to identify other bioactive components in MOAE.

## 5. Conclusions

In conclusion, we demonstrated that MOAE exhibits potent inhibitory activity against the proliferative and migration of A549 cells, and we showed that MOAE could induce apoptosis and cell cycle arrest in A549 cells. Collectively, our results suggested that these effects of MOAE may be mediated through the inhibition of JAK2/STAT3 signaling pathway activation.

## Data Availability

The data used to support the findings of this study are included within the article. Other data used to support the findings of this study are available from the corresponding author upon request.
